# Staging and haematological abnormalities of HIV-infected persons in Mangaung in the Free State Province, South Africa

**DOI:** 10.4102/phcfm.v4i1.462

**Published:** 2012-10-15

**Authors:** Andries J. Groenewald, Corinna M. Walsh, Hendrik J. van Wyk, Sanet van Zyl, Lynette J. van der Merwe

**Affiliations:** 1 Department of Chemical Pathology, University of the Free State, South Africa; 2 Department of Nutrition and Dietetics, University of the Free State, South Africa; 3 Department of Basic Medical Sciences, University of the Free State, South Africa

## Abstract

**Background:**

The prevalence of HIV in specific areas of South Africa and its impact on haematological parameters are largely unknown.

**Objectives:**

To (1) describe the prevalence of HIV, (2) stage HIV based on CD_4_ counts, and (3) determine haematological parameters amongst participants in Mangaung, Free State Province.

**Method:**

Blood specimens were obtained from 419 participants in formal and informal settlements in Mangaung. Participants were 25–64 years of age; 23.4% were male. Males and females were a mean of 45.1 and 44.3 years of age, respectively. Screening for HIV status was performed. Positive results were confirmed by a second test. Full blood counts were performed on all participants, and CD_4_ counts only on HIV-positive serum.

**Results:**

The overall prevalence of HIV was 40.8%. The highest prevalence occurred in the 31–40 years age group, with 38.4% of females and 39.5% of males being infected. More than 33% of HIV-uninfected participants were 51–60 years of age. No significant change in CD_4_ count with age was observed, probably because 19.1% of the 225 respondents who reported using medication were receiving antiretroviral (ARV) treatment. Haematological results showed that HIV-infected participants had significantly reduced values for haemoglobin, leukocytes, neutrophils and lymphocytes, compared to HIV-uninfected participants. The mean corpuscular volume (MCV) was significantly higher in HIV-infected participants.

**Conclusion:**

A high prevalence of HIV-infection was found. Anaemia and significantly reduced white blood cell counts were observed in the HIV-infected group. No significant change in CD_4_ counts with age was observed and could possibly be attributed to ARV therapy.

## Abstrait

**Staging et anomalies hématologiques chez des personnes séropositives à Mangaung dans la province de l’État libre, Afrique du Sud**

**Contexte:**

La prévalence du VIH dans des zones spécifiques d'Afrique du Sud et son impact sur les paramètres hématologiques sont pour l'essentiel inconnus.

**Objectifs:**

(1) Déterminer la prévalence du VIH, (2) le stade de progression du VIH sur la base de la numération des CD4 et (3) les paramètres hématologiques chez des participants à Mangaung, province de l’État libre.

**Méthode:**

Des échantillons sanguins ont été obtenus auprès de 419 participants dans des zones d'habitation formelles et informelles à Mangaung. Les participants étaient âgés de 25 à 64 ans et 23.4% étaient de sexe masculin. L’âge moyen des hommes et des femmes était de 45.1 er 44.3 ans respectivement. Un dépistage afin d’établir le statut VIH a été réalisé. Les résultats positifs ont été confirmés par un deuxième test. Une numération globulaire complète a été réalisée sur tous les participants, et une numération de CD4 uniquement sur les sérums diagnostiqués positifs pour le VIH.

**Résultats:**

La prévalence générale du VIH était de 40.8%. La prévalence la plus élevée s'est produite dans la tranche d’âge des 31 à 40 ans, 38.4% des femmes et 39.5% des hommes étant infectés. Plus de 33% des participants séronégatifs appartenaient à la tranche d’âge des 51 à 60 ans. Aucun changement significatif en termes de numération des CD4 en fonction de l’âge n'a été observé, probablement en raison du fait que 19.1% des 225 sondés ayant déclaré prendre des médicaments bénéficiaient d'un traitement antirétroviral (ARV). Les résultats hématologiques ont indiqué que les participants séropositifs présentaient des taux significativement réduits d'hémoglobines, de leucocytes, de neutrophiles et de lymphocytes, par rapport aux participants séronégatifs. Le volume globulaire moyen (VGM) était significativement plus élevé chez les participants séropositifs.

**Conclusions:**

Une prévalence élevée de l'infection par le VIH a été constatée. Une anémie et un nombre de globules blancs significativement réduit ont été observés dans le groupe des participants séropositifs. Aucun changement significatif en termes de numération des CD4 en fonction de l’âge n'a été observé, ce qui pourrait probablement être attribué à la thérapie ARV.

## Introduction

### Key focus

According to a national community-based survey conducted in South Africa in 2004, the prevalence of HIV in the general population at that time was 11.4%.^1^ In 2006, the prevalence of HIV infection amongst antenatal clinic attendees varied between 15.1% in the Western Cape and 39.1% in KwaZulu-Natal. At that time, the Free State Province had a reported prevalence of 31.1%, whilst the national prevalence was 29.1%.^2^

Staging is used to classify patients living with HIV and patients that have AIDS into groups with different prognoses. The Centres for Disease Control and Prevention (CDC)^3^ uses CD_4_ counts of > 500 cells per mm^3^, 200–499 cells per mm^3^ and < 200 cells permm^3^ to determine clinical and therapeutic management of HIV-positive adolescents and adults. A CD_4_ count of < 200 cells permm^3^ is regarded as indicative of progression towards full-blown AIDS.^3^

A routine full blood count can be valuable as part of the general investigation of an acute illness, regular monitoring of HIV infection, or monitoring of the side-effects of certain drug treatment regimens such as Zidovudine (AZT).^4^ Haematological abnormalities may give an indication of impaired oxygen-carrying capacity, risk of infections and bleeding tendencies.^4^

#### Aim of the study

This investigation formed part of the Assuring Health for All in the Free State (AHA FS) research project, a prospective epidemiological study with the main aim to determine how living in rural and urban areas affects lifestyle and indicators of health. A multidisciplinary research team investigated socio-demographic status; household food security; dietary intake; levels of physical activity; knowledge, attitudes and practices related to nutrition; and reported health status, using standardised questionnaires. In addition to a medical examination, anthropometric measurements and blood specimens were also obtained for various investigations. A rural baseline study was completed in 2007,^5^ whilst the urban baseline study was conducted in Mangaung in the Motheo District of the Free State province during 2009.

The aim of this particular component of the study was to, (1) describe the prevalence of HIV, (2) stage HIV by means of CD_4_ counts, and (3) determine haematological differences between HIV-uninfected and HIV-infected participants in urban Mangaung, and to compare the findings with data obtained from the rural settlements previously investigated.^5^

#### Significance

We determined the prevalence of HIV infection in an urban area in the Free State Province, staged HIV-infected participants into different CD_4_ count categories, and measured the extent of haematological abnormalities in all participants.

## Ethical considerations

Approval to conduct the study was obtained from the Ethics Committee of the Faculty of Health Sciences at the University of the Free State in Bloemfontein (ETOVS nr: 21/07), the provincial Department of Health and local municipalities. Before onset of the study, all selected households were visited by trained fieldworkers and written informed consent to participate was obtained in the language of choice. Participation was voluntary and participants could withdraw at any time. All information was treated as strictly confidential. Pre- and post-intervention counseling was provided by medical practitioners. After completion of the urban baseline study and according to their clinical findings, participants who required medical services for management and follow-up were referred to the appropriate local or provincial facilities.

## Methods

### Setting

The urban baseline study was performed over a ten-day period in March 2009. Fasting venous blood samples were obtained from participants from 391 black households (*n* = 419), living in formal and informal settlements in Mangaung, Bloemfontein.

### Sampling and design

The number of plots in the Mangaung University Community Partnership Programme (MUCPP) service area was counted on a municipal map and included Buffer, Freedom Square, Kagisanong, Chris Hani, Namibia and Turflaagte. An estimate was made of additional squatter households in open areas. A stratified proportional cluster sample was selected, stratified by area and formal plot and/or squatter households in open areas. Using randomly selected X and Y coordinates, 100 starting points were selected in this way. This sample was considered representative of the population served by the MUCPP clinic and results are indicative of the extent of the problem in this area. From each starting point, five adjacent starting households were approached. All volunteers between the ages of 25 and 64 years were eligible to participate. With the exception of age, the only requirement for inclusion in the study was written informed consent.

### Procedure

Final-year and postgraduate students from the Department of Nutrition and Dietetics at the University of the Free State (UFS) conducted the interviews to complete household socio-demographic and individual health questionnaires. The interviews were performed under supervision of lecturers as part of the Service Learning function of the University.

Medical examinations were performed at the MUCCP clinic by medical doctors from the Department of Basic Medical Sciences, Faculty of Health Sciences, UFS. It is possible that participants with an existing medical problem were more likely to participate in the study, whilst bed-ridden participants could have found it difficult to visit the research venue to participate. More women than men participated in the study, most probably because more men are employed. Due to these reasons, the authors acknowledge that the study group is probably not representative of the general population.

All participants were screened for HIV using two fourth-generation serum assays. Primary screening for HIV status was performed using the Enzygnost HIV Integral II Ag/Ab test (Dade Behring; Marburg, Germany). Positive cases were confirmed by the Vironostica HIV Uni-Form II Ag/Ab test (bioMérieux; Boxtel, The Netherlands). Serum CD_4_ counts were measured with an Epics XL flow cytometer (Beckman Coulter; Atlanta, GA, USA). Full blood counts were obtained from blood collected in EDTA-containing tubes, using a Sysmex XT 2000i analyser (Roche Diagnostics; Indianapolis, IN, USA).

### Analysing

Descriptive statistics were used to report findings, and the *t*-test to measure the significance of differences (*p* ≤ 0.05) between HIV-infected and -uninfected groups.

## Results

Blood specimens were obtained from 419 participants in formal and informal settlements in Mangaung. Participants between 25 and 64 years of age were included in the study, of which 23.4% were male (mean age 45.1 years) and 76.6% female (mean age 44.3 years).

Of the 225 respondents who reported using medication, 43 (19.1%) indicated that they were receiving antiretroviral (ARV) treatment at the time of the study.

The overall prevalence of HIV infection in this survey was 40.8%, with 41.7% of female and 38.5% of male participants being infected. The peak prevalence of HIV occurred in the 31–40 years age group, with 38.4% of females and 39.5% of males in this age group being HIV-infected.

The distribution of CD_4_ counts, performed only on HIV-infected participants, showed that 22.2%, 42.1% and 35.7% of participants had CD_4_ counts above 500 cells/mm^3^, between 200 and 499 cells/mm^3^, and below 200 cells/mm^3^, respectively.

**FIGURE 1:**
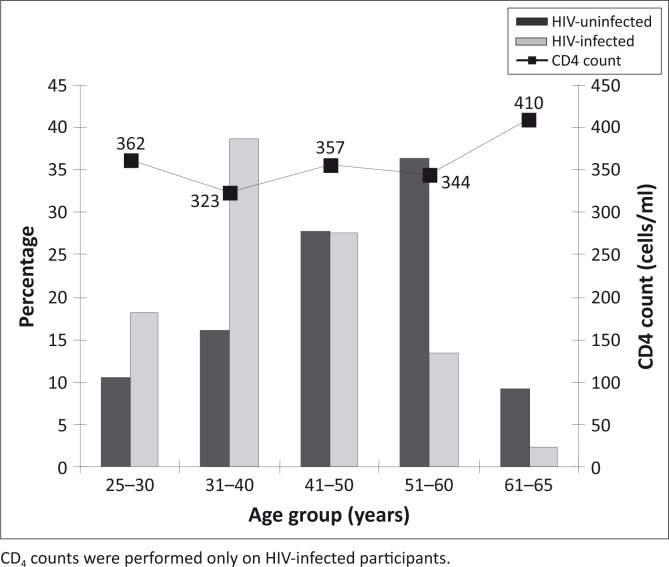
Distribution of HIV infection and mean CD_4_ counts according to age groups in urban Mangaung.

The age distribution of HIV-uninfected and HIV-infected participants and the distribution of mean CD_4_ counts in relation to age (Figure 1). Participants were grouped in 10-year intervals. The majority (38.6%) of HIV-infected participants were 31–40 years of age, whilst HIV-uninfected individuals were mostly represented in the 51–60 years age group (36.3%). No significant change in CD_4_ count with age was observed.

Haematological results showed that HIV-infected participants had significantly reduced haemoglobin (in both males and females *p* < 0.0001), leukocyte (*p* < 0.0001), neutrophil (*p* < 0.0001) and lymphocyte values (*p* < 0.0001) compared to HIV-uninfected participants. The mean corpuscular volume (MCV) was significantly higher (*p* < 0.001) in HIV-infected participants. No significant difference was observed between the two groups with regard to the platelet count. Results of haematological investigations are presented (Table 1).

**TABLE 1:** Haematological findings in HIV-infected and HIV-uninfected participants in Rural‡ and urban areas of the Free State.

Haematological variables	Normal range†	Rural‡					Urban				
		
		Mean HIV-negative	*n* = 458	Mean HIV-positive	*n* = 94	*p*	Mean HIV-negative	*n* = 248	Mean HIV-positive	*n* = 171	*p*
Male Haemoglobin (g/dL)	14.3–18.3	15.3	1.3	14.4	1.7	< 0.01	15.2	1.5	13.7	2.1	< 0.0001
Female Haemoglobin (g/dL)	12.3–16.3	13.9	1.3	13	1.3	< 0.0001	13.5	1.2	12.4	1.8	< 0.0001
White blood cell count (x 10^9^ cells/L)	4–10	7.4	2.2	6.5	2.6	< 0.001	6.5	2.0	5.4	1.8	< 0.0001
Mean corpuscular volume (fL)	80–100	94.3	6.7	93.6	7.2	> 0.05	91.2	6.2	93.9	8.4	< 0.001
Platelet count (x 10^9^ cells/L)	137–373	278.6	74.0	269.2	72	> 0.05	279.4	81.0	267	88.0	> 0.05
Neutrophil count (x 10^9^ cells/L)	2–7.5	4.0	1.8	3.4	2.1	< 0.01	3.5	1.6	2.8	1.3	< 0.0001
Lymphocyte count (x 10^9^ cells/L)	1–4	2.7	0.9	2.4	1.1	< 0.01	2.4	0.8	2.0	0.8	< 0.0001

*n*, Given as number; *p*, significant value.

† Normal ranges used by the laboratories of the South African National Health Laboratory Service (NHLS).

‡ Information obtained from the following article: Groenewald AJ, Van Wyk HJ, Van Zyl S, Van der Merwe LJ, Walsh CM. Staging and haematological abnormalities of HIV-infected persons in the rural Free State Province of South Africa. Afr J Prim Health Care Fam Med. 2011;3(1). http://dx.doi.org/10.4102/phcfm.v3i1.222

## Discussion

The Mangaung area consists of both formal and informal settlements. The prevalence of HIV infection (40.8%) confirms a recent finding^6^ in which 60% of women aged 25–34 years and 38% of women aged 35–44 years were found to be HIV-positive. This prevalence of HIV in the Mangaung area is much higher than the observed 17.1% amongst participants in rural the Free State, which included Springfontein, Trompsburg, and Philippolis.^5^

Nationally, HIV prevalence peaks in women aged 20–29 years (24.1%) and in men aged 30–39 years (21.3%).^1^ In a rural study,^5^ it was observed that HIV prevalence peaked later at 31–40 years (41.3%) in women and 41–50 years (37.9%) in men. The observation that peak prevalence of HIV infection in women occurs at a younger age than in men could reflect the fact that men tend to have sexual partners younger than themselves.^7^ In the present (urban) study, HIV prevalence in both females (38.4%) and males (39.5%) peaked at 31–40 years. This high urban prevalence of HIV where men peaked earlier and at the same time as women may be due to the effect of lifestyle transitions that occur when people move from rural to urban areas.

CD_4_ count and viral load tests are essential parts of the monitoring of both the course of HIV infection over time as well as the patient's response to treatment.^8^ CD_4_ counts greater than 500 cells/mm^3^ are associated with a healthy immune system, which weakens with progression of HIV infection until levels lower than 200 cells/mm^3^ are reached.^3^ Low CD_4_ counts are associated with a compromised immune system, serious infections and general health problems. In a recent rural study,^5^ the peak prevalence of HIV infection in the age group 31–40 years supported the significantly low mean CD_4_ count (276 cells/mm^3^) in the age group 41–50 years. This finding suggests that from the onset of infection with the virus until progression into AIDS, takes approximately ten years.^9^ In the present study, the prevalence of HIV also peaked at 31–40 years (Figure 1). Although not statistically significant, CD_4_ counts were generally much lower in the urban (mean ± s.d. = 346 ± 251 cells/mm^3^) than in the rural^5^ (mean ± s.d. = 399 ± 270 cells/mm^3^) participants. No significant change with age was observed, probably because a large number of participants received antiretroviral (ARV) treatment.

Antiretroviral treatment may cause macrocytosis^10^ and may be the reason why the mean corpuscular volume of HIV-infected participants in this urban study was significantly higher than in uninfected participants (*p* > 0.001, Table 1). In the rural study, the mean corpuscular volume remained unchanged, as none of the participants received ARV treatment.^5^

Differences in the rest of the haematological parameters between HIV-infected and -uninfected individuals (Table 1) were in general more pronounced in urban participants than found in a recent rural study.^5^ Haemoglobin was significantly reduced in HIV-infected males (*p* < 0.0001) and females (*p* < 0.0001) compared to uninfected participants (Table 1). Anaemia could contribute to symptoms of fatigue and breathlessness. It is more common amongst people with HIV infection and may be caused by HIV itself, opportunistic infections or treatment.^4^ The significantly reduced haemoglobin values found in HIV-uninfected males are probably indicative of a generally ill study population.

Total as well as differential white cell counts were determined for each participant (Table 1). Compared to HIV-uninfected participants, significantly reduced white blood cell (*p* < 0.0001) and neutrophil counts (*p* < 0.0001) might enhance the risk of bacterial and fungal infections.^4^ Significantly reduced lymphocyte counts (*p* < 0.0001) observed in our study, may be associated with HIV-related infection and killing of CD_4_ T-cells.^8^ Lymphocyte counts between 1.0 and 2.0 x 10^9^ cells/L were found to be a significant predictor of CD_4_ < 200 cells mm^-3^.^11^ Our mean (± s.d.) lymphocyte count for participants with CD_4_ < 200 cells/mm^3^ was 1.45 (± 0.66) in urban participants. Lymphocyte count may therefore serve as a useful predictive tool in the management and monitoring of HIV-infected patients in resource-limited settings.^11^ The impact of HIV-infection on haematological changes is reversible by highly active anti-retroviral therapy (HAART).^12^

## Limitations of the study

A possible limitation of the study could be that participants with a specific medical problem were more likely to participate in the study. More women than men participated in the study, most probably because more men were employed. Due to these factors, we acknowledge that the study group was probably not representative of the general population.

### Recommendations

Only 19.1% of HIV-infected participants indicated that they were receiving ARV treatment. Yet, 35.7% of HIV-infected participants had a CD4 count below 200 cells/mm3. ARV treatment for more participants would thus seem justified and is recommended.

## Conclusion

The prevalence of HIV in urban Mangaung was higher compared to the rural Free State. A high prevalence of anaemia and significantly reduced white blood cell and neutrophil counts were observed in HIV-infected participants. The CD_4_ counts were in general much lower than in the rural study.^5^ No significant change in CD_4_ counts with age was observed, probably because a large number of participants received ARV treatment. The latter may also cause macrocytosis,^10^ possibly reflected by the mean corpuscular volume being significantly larger in HIV-infected participants. All these changes are probably the result of a change in lifestyle and ARV treatment.
